# The Secretomes of *Aspergillus japonicus* and *Aspergillus terreus* Supplement the Rovabio^®^ Enzyme Cocktail for the Degradation of Soybean Meal for Animal Feed

**DOI:** 10.3390/jof7040278

**Published:** 2021-04-07

**Authors:** Delphine Grandmontagne, David Navarro, Virginie Neugnot-Roux, Simon Ladevèze, Jean-Guy Berrin

**Affiliations:** 1 INRAE, Aix Marseille University, BBF, UMR1163, 13009 Marseille, France; delphine.dessaint@univ-amu.fr (D.G.); david.navarro@inrae.fr (D.N.); sl854@cam.ac.uk (S.L.); 2 ADISSEO, 135 Avenue de Rangueil, INSA Toulouse, Hall Gilbert Durand, 31400 Toulouse, France; virginie.neugnot-roux@adisseo.com; 3 CIRM-CF (Centre International des Ressources Microbiennes- Champignons Filamenteux), INRAE, Aix Marseille University, 13009 Marseille, France; 4 University of Cambridge, Sanger Building, 80 Tennis Ct Rd, Cambridge CB2 1GA, UK

**Keywords:** fungal diversity, filamentous fungi, *Aspergillus*, fungal secretomes, soybean meal, enzymes, animal feed

## Abstract

One of the challenges of the 21st century will be to feed more than 10 billion people by 2050. In animal feed, one of the promising approaches is to use agriculture by-products such as soybean meal as it represents a rich source of proteins. However, soybean meal proteins are embedded in a complex plant cell wall matrix, mostly composed of pectic polysaccharides, which are recalcitrant to digestion for animals and can cause digestive disorders in poultry breeding. In this study, we explored fungal diversity to find enzymes acting on soybean meal components. An exploration of almost 50 fungal strains enabled the identification of two strains (*Aspergillus terreus* and *Aspergillus japonicus*), which improved the solubilization of soybean meal in terms of polysaccharides and proteins. The two *Aspergilli* strains identified in the frame of this study offer a promising solution to process industrial food coproducts into suitable animal feed solutions.

## 1. Introduction

Since the middle of the 20th century, the human population has tripled, reaching 7 billion today and most probably 10 billion by 2050 [1,2,3]. In order to maintain access to nutrition, a sustainable production of cereals and meat is essential. Feeding animals represents about 60–70% of the price in poultry production system. In the current context of the development of a circular economy, a promising approach is to feed animals with agriculture by-products such as rapeseed, canola, sunflower, and soybean meal (SBM) [4]. These bioresources are complex: mostly composed of polysaccharides and proteins embedded in a plant cell wall matrix. The rich amino acid value of SBM makes it very attractive for poultry and swine industries [5]. SBM is composed of 44–50% crude proteins, 3% crude fat, and about 35% carbohydrates [6]. Cell wall carbohydrates contain 10% of free sugars (sucrose, stachyose, and raffinose), 8% of cellulose, and 17% of pectins (mainly rhamnogalacturonan I (RGI), arabinogalactan, and xylogalacturonan polymers) [7]. Even though carbohydrates are the most important energy sources for nonruminant and ruminant animals [8], SBM is not digestible by poultry since their digestive tracts do not display the endogenous enzymes required to degrade these polysaccharides [9]. Like most of the agricultural by-products used in animal feed, SBM need to be bioprocessed to become digestible [10], as it can lead to digestive disorder, gas accumulation, diarrhea, and lack of nutrients [11], thus decreasing the yield of meat production.

Addition of exogenous enzymes in animal diets can increase the production of meat per animal, thereby decreasing the time and cost of production [12]. Since the 1980s, exogenous enzymes (cellulases, phytases, xylanases, pectinases, and proteases) have been commonly used to supplement endogenous enzymes secreted by the digestive tract of the animal [9,13,14,15,16]. Most of the exogenous enzymes used in animal feed are of fungal origin. Indeed, the use of fungi in the degradation of plant raw material is well recognized as they secrete a rich panel of enzymes (i.e., secretome) able to degrade complex biomass [17,18,19,20,21,22]. Fungi can adapt their secretomes depending on environmental conditions, including the growth substrate, temperature, and growth phases [20,23]. These fungal secretomes have allowed the development of industrial enzymatic cocktails, including in the animal feed field. The Rovabio^®^ is an enzymatic cocktail produced from the ascomycete fungus *Talaromyces versatilis* [24]. The Rovabio^®^ cocktail contains more than 200 different enzymes, mostly carbohydrate-active enzymes (CAZymes; [25]), which target the different components of plant cell wall, cellulose, and hemicelluloses [26]. This cocktail, which is efficient for enhancing broiler feed digestibility [27,28,29,30,31], was improved using GH62 arabinofuranosidases [27,32] and GH10 and GH11 xylanases [33,34] to increase its ability to degrade recalcitrant nonstarch polysaccharides (i.e., xylans). However, for pectin-rich biomasses, especially SBM, there is a possibility to improve established enzymatic cocktails.

In this study, fungal diversity was explored, without a priori knowledge, as a source of enzymes for SBM degradation. Exploration of fungal diversity is a relevant approach to find enzymes of interest for many biotechnological applications [17,35,36]. Herein, the potential of fungal strains to grow on SBM was evaluated to select a few strains of interest. Several fungal secretomes were produced, and their efficiency was assessed using a simple method to easily measure SBM solubilization as well as proteins and sugars solubilization.

## 2. Material and Methods

### 2.1. Fungal Strains

Fungal strains were from the CIRM-CF (Centre International de Ressources Microbiennes-Champignons Filamenteux, INRAE, Marseille, France) collection. All strains were authenticated using classical taxonomy, enzymatic characterization, and ITS (internal transcribed spacer) molecular tools [37] as previously described [17].

Forty-nine fungal strains were selected from the CIRM-CF collection. They represent a wide diversity with 33 different families belonging to the basidiomycetes (29 strains), ascomycetes (16 strains), and mucuromycetes (four strains) (Table 1). Although all these fungi are not known to be animal pathogens, we carefully checked the literature to avoid any known production of antibiotics or mycotoxins that could represent a potential risk for the health of animals.

Each fungal strain was incubated on different solid media: a minimum control medium containing only agar and a rich control medium (containing potato dextrose agar for ascomycetes and mucoromycetes strains, and malt-agar medium (malt extract, 2% *w*/*v*) for basidiomycetes). Two other media with 15 g/L SBM (based on dry matter) were produced. The first one contained agar and micronized raw SBM, and the second one contained agar and recalcitrant micronized SBM. Recalcitrant micronized SBM was prepared by hydrolysis of SBM with the Rovabio^®^ enzymatic cocktail. For this, 120 g of rough SBM were hydrolyzed by 8 mL of Rovabio^®^ (90 mg of protein per mL) for 24 h, at 37 °C, 130 rpm in sodium acetate buffer (100 mM, pH 4). After hydrolysis, recalcitrant SBM was washed with water twice and finally dried for 48 h at 60 °C. Then, the SBM was ball-milled to obtain 40 g of micronized SBM powder (below 100 µm particle size). Fungi were incubated at 25 °C. At 5, 8, and 11 days of growth, diameter and growth density were measured. The density of the mycelium was evaluated using scores ranging from 1 to 5, by comparison of mycelium densities to the control media (score of 1 when equivalent to poor control media and score of 5 when equivalent to rich control media).

### 2.2. Culture Conditions and Secretome Preparation

Fungal secretomes were prepared based on previous observations [38]. Selected fungal strains were cultured on rich agar media for 10 days to produce their secretomes. The spores of the sporulating fungi were used directly to inoculate the culture media (2 × 10^5^ spores/mL). Basidiomycetes need an extra Roux’s flask step. The Roux’s flask medium was composed of yeast extract (20 g/L), bactopeptone (40 g/L), and glucose (100 g/L). When the fungal mycelium covered the surface of Roux’s flask (between 10 and 50 days depending on the strains), fungi were filtered and mixed to inoculate the media (between 400 and 600 mg of fungal biomass per liter of media). Fungal cultures were grown in 500 mL baffled flasks, in a 100 mL liquid medium containing 15 g/L (based on the dry matter) of autoclaved biomass (cellulose, micronized sugar beet pulp (SBP) or micronized SBM) as a carbon source, 2.5 g/L of maltose as a starter, 1.842 g/L of diammonium tartrate as a nitrogen source, 0.5 g/L yeast extract, and salts (KH_2_PO_4_: 0.2 g/L; CaCl_2_: 0.0132 g/L; MgSO_4_: 0.5 g/L). Flasks were incubated at 30 °C, with orbital shaking at 120 rpm. After 7 days of inoculation, the culture broths (secretomes) were harvested and pooled (total volume 300 mL per condition). Supernatants were filtered through a 10-kDa pore-sized membrane (Vivaspin polyethersulfone, Sartorius, Göttingen, Germany), diafiltered in sodium acetate buffer (50 mM, pH 5), and concentrated to a final volume of 10 mL. Secretomes were finally aliquoted and stored at −20 °C for further use. The protein content of each secretome was analyzed using a 10% Tris-glycine precast SDS-PAGE stain-free gel (Bio-rad, Marnes-la-Coquette, France) stained using Coomassie blue. The molecular mass under denaturing conditions was determined with PageRuler prestained protein ladder (Thermo Fisher Scientific, Bartlett, IL, USA).

### 2.3. Liquid Fermentation in Bioreactor Conditions

*Aspergillus terreus* cultures were performed in 2 liter bioreactors, containing a 1.5 liter liquid medium based on 15 g/L (based on the dry matter) of autoclaved biomass (micronized or 3 mm particles sized SBP) as a carbon source, 2.5 g/L of maltose as a starter, 1.842 g/L of diammonium tartrate as a nitrogen source, 0.5 g/L yeast extract, salts (KH_2_PO_4_: 0.2 g/L; CaCl_2_: 0.0132 g/L; MgSO_4_: 0.5 g/L), 0.05 g/L Tween 80, and anti-foam. Bioreactors were incubated at 30 °C, with 8 L/h O_2_ and orbital shaking at 120 rpm (marine propellers). After 7/10 days of inoculation, secretomes were harvested. Supernatants were then filtered on a 10-kDa pore-sized membrane (Vivaspin polyethersulfone, Sartorius) and filtered dialyzed in sodium acetate buffer (pH 5, 50 mM) and concentrated to a final volume of 10 mL. They were aliquoted and stored at −20 °C for further use.

### 2.4. Enzymatic Degradation of Soybean Meal

Micronized SBM (150 mg) was treated with the secretomes (0.1 mg of proteins) of selected fungi in 2 mL Eppendorf tubes. Secretomes were diluted in 1 mL sodium acetate buffer (100 mM, pH 4). The samples were incubated on an orbital shaker at 850 rpm for 24 h at 37 °C. Reactions were stopped by addition of 1 mL of KOH 0.34% (*w*/*v*) and incubated for 20 min on a digital tube revolver (ThermoFisher scientific, Waltham, MA, USA) at 20 rpm. Samples were then centrifuged at 4 °C, 11,000 g for 15 min, and supernatants were collected to perform DNS, TNBS, and Bradford assays (see below). The pellets were washed three times with water and dried at 100 °C to perform dry matter measurements and deduce SBM solubilization.

The reducing sugars released were quantified using the dinitrosalicylic acid (DNS) assay [39], proteins released (Araba and Dale, 1990) were quantified using the Bradford assay [40] and the TNBS (2, 4, 6-Trinitrobenzenesulfonic acid) assay [41], respectively.

The commercial enzymes used were Rovabio^®^ Advance (Adisseo, Toulouse, France) and a preparation serine-protease (DSM, Village-Neuf, France).

### 2.5. Sugar Composition

To evaluate the monomeric sugar composition of polysaccharides, pellets were hydrolyzed with sulfuric acid (76% (*v*/*v*), 30 min, 30 °C). Another sulfuric acid hydrolysis was performed (1 M, 100 °C, 2 h). Neutral sugars derivatization was performed with alditol acetate and analyzed on gas-phase chromatography [42]. Acidic sugars were evaluated with the Skalar system, using the colorimetric MHDP method [43].

## 3. Results and Discussion

### 3.1. Exploration of Fungal Biodiversity

The ability of filamentous fungi to degrade lignocellulosic biomass has been studied in a range of basidiomycetes and ascomycetes [17,20,38] but to our knowledge, no large screening has been performed using SBM as substrate. Here, four media were used to test the ability of fungi isolates to grow on SBM. Among these four media, two were used as controls to confirm the capacity of each fungal strain to grow on the MA2 or PDA media (rich media) while not being able to do so on the agar media (poor medium). The two other media contained micronized SBM and were prepared to test the ability of each fungal strain to grow on SBM. To get a more selective substrate, we prepared a “recalcitrant SBM” by hydrolyzing it using the Rovabio^®^ enzyme cocktail.

**Figure 1:**
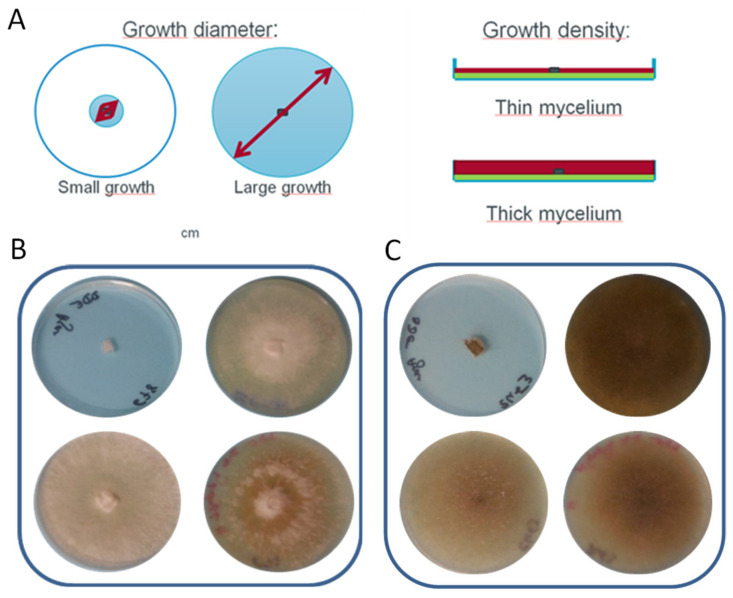
Evaluation of the fungal growth on soybean meal. Panel (**A**): schematic description of the parameters used to select the best-performing strains. Panels (**B**,**C**): examples with growth of *Oxyporus latemarginatus* BRFM 678 (**B**) and *Absidia glauca* BRFM 2463 (**C**). composition of the petri dishes: from the top left-hand corner to the bottom right-hand corner: agar medium, PDA or MA2 medium, soybean meal (SBM)-composed medium, recalcitrant SBM-composed medium.

Out of the 49 isolates, 44 strains were able to grow on SBM and recalcitrant SBM substrates with a growth diameter larger than 2 cm. The selection was done after 8 days of growth, as it was the midterm growth for most of fungi under our experimental conditions (Supplementary Materials Table S1). To measure fungal growth, two parameters were used: the growth diameter and density of the mycelium (Figure 1). Growth diameters were measured in centimeters. As fungi are able to produce filaments to explore their environment without degrading the substrate, the thickness of these filaments was evaluated by comparison to the controls (poor and rich medium control plates) and described as “density” of the growth.

### 3.2. Selection of the Fungal Strains

None of the 49 strains tested grew on the agar medium, while a thick and abundant mycelium was observed in the rich medium condition. The selection criteria were a diameter wider than 4 cm and a density above 3 (corresponding to an intermediate filamentous density between the filamentous density obtained on both the poor medium and the rich media) on SBM or/and recalcitrant SBM media (Supplementary Materials Table S1). Using these criteria, we selected 14 strains, among which 7 were basidiomycetes: *Dichostereum effuscatum* (BRFM 91); *Lentinula edodes* (BRFM 353); *Oxysporus latemarginatus* (BRFM 678); *Pleurotus ostreatus* (BRFM 853); *Pycnoporus sanguineus* (BRFM 902); *Gymnopilus junonius* (BRFM 969); *Amauroderma calcigenum* (BRFM 1190); two mucoromycetes: *Rhizopus arrhizus var. arrhizus* (BRFM 1095); *Absidia glauca* (BRFM 2463); and five ascomycetes: *Nectria pseudotrichia* (BRFM 1017); *Neurospora crassa* (BRFM 1092); *Pestalotiopsis* sp. (BRFM 1648); *Aspergillus japonicus* (BRFM 405); and *Aspergillus terreus* (BRFM 111).

### 3.3. Fungal Secretomes Production

The next step was to investigate the enzymes secreted by these fungal strains upon growth on SBM. We decided to further characterize only the strains for which genomic data were available to facilitate future postgenomic analyses. Therefore, we restricted our study to five strains: *A. glauca* (BRFM 2463), *G. junonius* (BRFM 969), *A. japonicus* (BRFM 405), *A. terreus* (BRFM 111), and *L. edodes* (BRFM 353).

Fungal enzymes secretion is regulated by the type and the complexity of the substrate used as inducer in the culture [20,38,39]. In this study, we selected three different types of plant biomass: cellulose, SBP, and SBM as they differ in terms of composition. While cellulose is widely used as an inducer to favor the secretion of fungal CAZymes, SBP is rich in pectin, and SBM is our biomass of interest. All the fungal isolates tested were able to grow on these three substrates with a satisfactory yield of protein secretion (Table S1). After 7 days of growth, fungal cultures were harvested and secretomes were filtered, dialyzed, and analyzed using SDS-PAGE, revealing a diversity of secreted protein profiles (Figure S1).

### 3.4. Development of a Simple Method to Assess the Efficiency of Each Secretome to Degrade Soybean Meal

**Figure 2:**
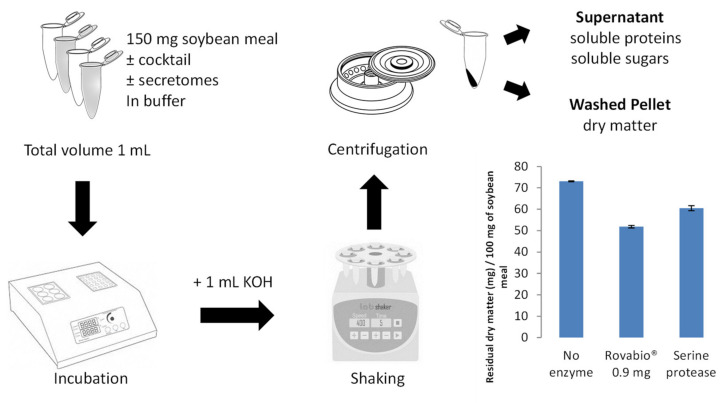
Schematic representation of the method to assay SBM degradation. The bar chart shows the method validation using the Rovabio^®^ enzymatic cocktail (0.9 mg of enzyme) and a serine-protease preparation (10 mg). Error bars indicate standard deviations of triplicate independent experiments.

The objective was to develop a simple method to allow parallelization of fungal secretomes analyses (Figure 2). The Rovabio^®^ enzyme cocktail containing CAZymes and serine proteases were used to develop the method. The reaction volume was set at 2 mL and the SBM quantity to 150 mg per assay using SBM micronized to a diameter below 100 µm (Figure 2). At this scale, we were able to confidently evaluate SBM degradation (Figure 2). Indeed, the low standard deviations could be considered a global measure of the reproducibility of each step of the method (substrate micronization, distribution, and incubation). This new method enabled to envisage the evaluation of fungal secretomes potential alone or in combination with the Rovabio^®^ cocktail.

### 3.5. Hydrolysis of Soybean Meal with Fungal Secretomes

**Figure 3:**
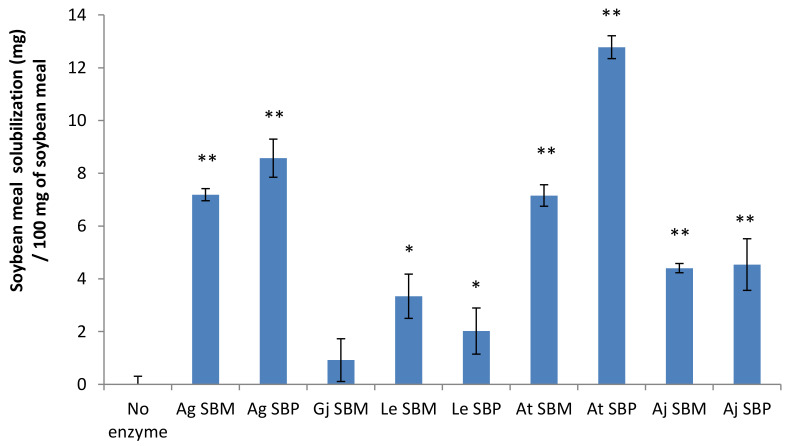
Ability of each secretome to hydrolyze soybean meal. Soybean meal solubilization was deduced from dry matter measurements (see Material and Methods). For each condition, 0.1 mg of each secretome’s enzymes was added. Ag: *Absidia glauca*, Gj: *Gymnopilus junonius*, Le: *Lentinula edodes*, At: *Aspergillus terreus*, and Aj: *Aspergillus japonicus.* Of note, Gj SBP data are missing as the secretome was too viscous and could not be collected and processed. Error bars indicate standard deviations of triplicate independent experiments. Significance of the results between “No enzyme” and secretome addition was assessed using *t*-test (*n* = 3) with *p*-value: *, *p*-value < 0.05; ** *p*-value < 0.01.

The ability of each secretome to hydrolyze SBM was first evaluated by measuring the residual dry matter before and after the action of each fungal secretome (Figure 3).

**Figure 4:**
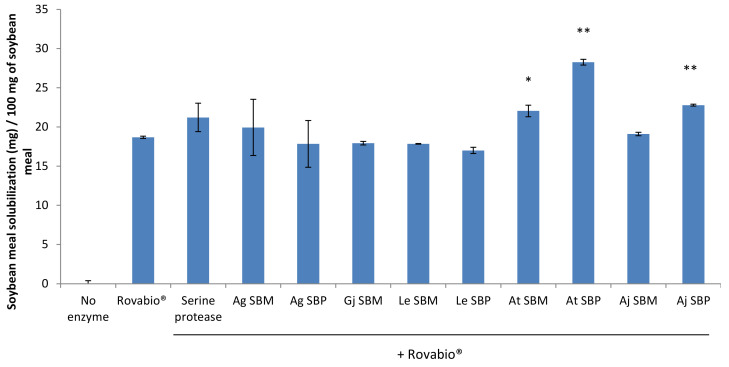
Ability of each secretome to supplement the Rovabio^®^ enzymatic cocktail. Rovabio^®^ (0.9 mg of enzyme) was supplemented with 0.4 mg of enzyme from the produced secretomes. Ag: *Absidia glauca*, Gj: *Gymnopilus junonius,* Le: *Lentinula edodes*, At: *Aspergillus terreus*, Aj: Aspergillus japonicus, SBP: sugar beet pulp, and SBM: soybean meal. Gj SBP data are missing as the secretome was too viscous and could not be collected and processed. Error bars indicate standard deviations of triplicate independent experiments. Significance of the results between Rovabio^®^ condition and Rovabio^®^ supplementation with secretome was assessed using *t*-test (*n* = 3) with *p*-value: *, *p*-value < 0.05; ** *p*-value < 0.01.

Although all the fungal strains were able to grow on SBM plates, only the secretomes of *Absidia glauca, Aspergillus terreus* and *Aspergillus japonicus* grown on SBM and SBP (and to a lesser extent *Lentinula edodes*) were able to significantly solubilize the SBM. As the objective of this study was to upgrade the Rovabio^®^ cocktail, we first assessed the amount of Rovabio^®^ enzymes to use in our assays. We established a relationship between the SBM solubilization and the amount of enzymes added for hydrolysis. In order to test whether the secretomes are able to supplement Rovabio^®^, the dose of Rovabio^®^ should not be chosen as a stopgap to the degradation of the meal. For this reason, a dose of 0.9 mg of enzyme was selected for the rest of the study (Figure S2). The supplementation of the Rovabio^®^ enzymatic cocktail was then performed with the nine secretomes produced (Figure 4). Out of the nine secretomes tested, significant improvements were observed with the *A. terreus* and *A. japonicus* secretomes. The most important improvement was obtained with the secretome of *A. terreus* and led to a significant increase of 28 mg of SBM solubilization. Overall, SBM inducer was not as efficient as SBP to produce a secretome able to supplement Rovabio^®^ enzymatic cocktail. The choice of the inductor is therefore crucial for the production of functional secretomes.

As both *A. terreus* and *A. japonicus* were able to supplement Rovabio^®^, we investigated the ability of other strains from the genus *Aspergillus* to supplement Rovabio^®^. We performed the same SBM hydrolysis assays with *A. niger* (BRFM 280), *A. brasiliensis* (BRFM 103), *A. tubenginsis* (BRFM 1521), *A. japonicus* (BRFM 405), and *A. terreus* (BRFM 111). We observed that all the *Aspergilli* strains are not able to supplement the Rovabio^®^ (Figure S3).

### 3.6. Effect of the Aspergilli Secretomes on the Release of Proteins and Sugars from Soybean Meal

**Figure 5:**
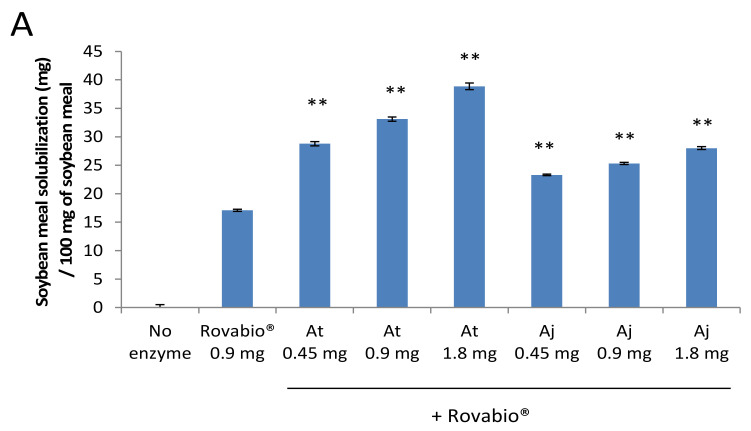

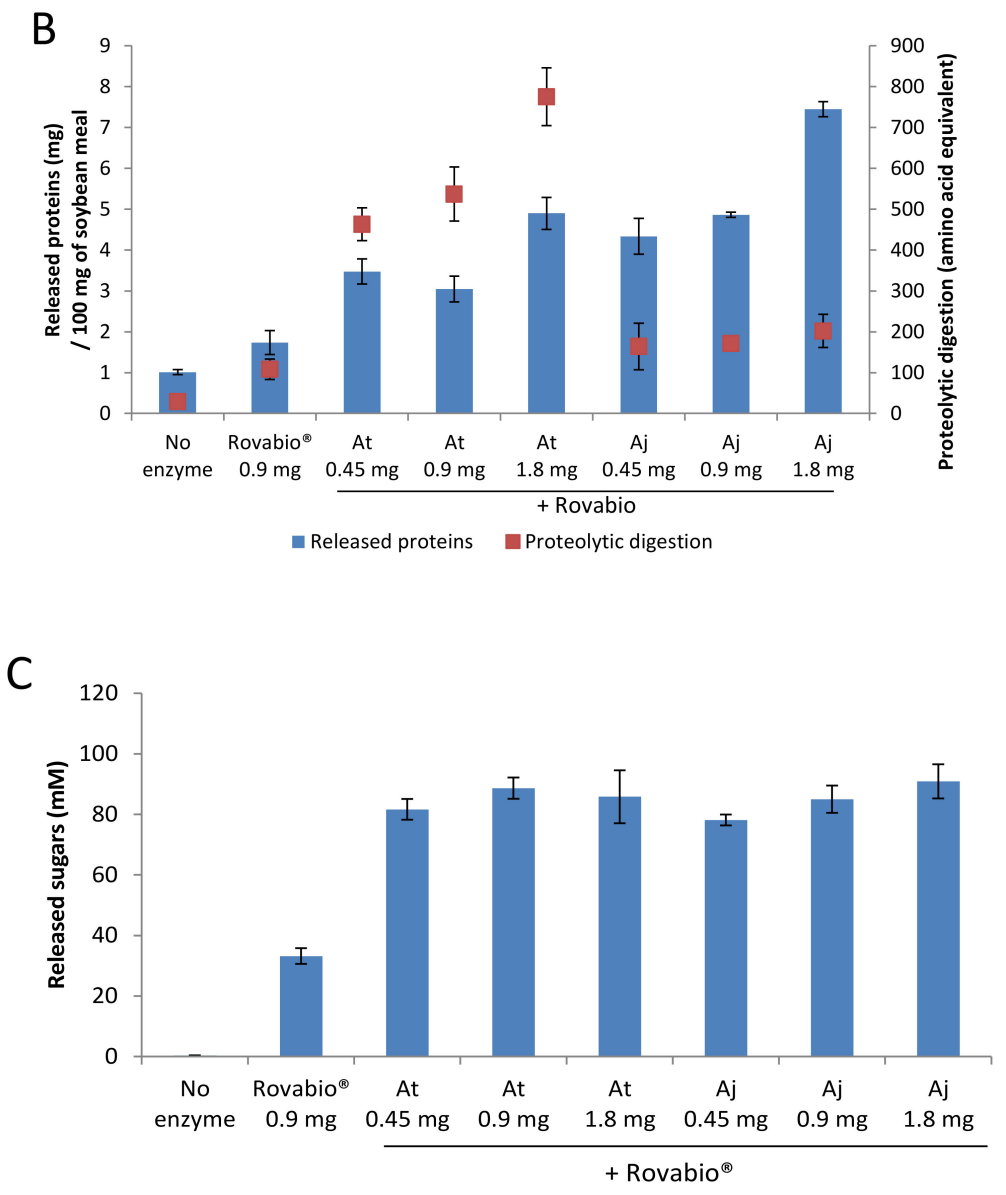
Effect of the *Aspergilli* secretomes on the protein and sugar release from soybean meal. Panel (**A**): soybean meal solubilization after supplementation of Rovabio^®^ with different amounts of enzymes for *Aspergillus terreus* (At) and *Aspergillus japonicus* (Aj) secretomes. Panel (**B**): total amount of released proteins (blue) and proteolytic digestion (red) after supplementation of Rovabio^®^ with different amounts of enzymes for *Aspergillus* secretomes. Panel (**C**): total amount of released sugars after supplementation of Rovabio^®^ with different amounts of enzymes from *Aspergillus* secretomes. Error bars indicate standard deviations of triplicate independent experiments. The significance of the results between Rovabio^®^ condition and Rovabio^®^ supplementation with secretome was assessed using *t*-test (*n* = 3). The *p*-value (**) obtained was <0.01 for all the results except for proteolytic digestion in the case of Rovabio^®^ supplemented with 0.45 mg Aj Panel (**B**).

To better understand the effect of *A. japonicus* (BRFM 405) and *A. terreus* (BRFM 111) secretomes on SBM, several complementary methods were used. The measurements of soluble sugars and proteins released were performed using the DNS and Bradford assays, respectively (Figure 5). A measurement of protein cleavage was also preformed using the TNBS method to evaluate proteolytic digestion (Figure 5). Following the addition of increasing amounts of enzymes from each secretome, the SBM solubilization gradually increased (Figure 5A). The boosting effect was the most significant with the secretome of *A. terreus* (1.8 mg), leading to 39% of solubilized matter compared to 17% when Rovabio^®^ was used alone. Supplementation of Rovabio^®^ with the different *Aspergilli* secretomes improved the solubilization of proteins. The TNBS assay revealed that the *A. terreus* secretome cleaves soybean proteins into smaller peptides compared to the *A. japonicus* secretome (Figure 5B). The supplementation of Rovabio^®^ with the different *Aspergilli* secretomes also increased the release of soluble sugars (Figure 5C). Although the DNS method can evaluate the reducing ends, it does not give any information about the length of the released sugars. Even though the increase observed in soluble sugars released suggests that the number of cleavages in soybean polysaccharides is higher in supplemented conditions, it does not give any information about the nature of the sugars.

Therefore, we performed a compositional analysis of the sugar monomers (rhamnose, fucose, arabinose, xylose, mannose, galactose, glucose, and uronic acids) present in the residual fraction of the SBM after enzymatic hydrolysis by Rovabio^®^ supplemented or not by *A. japonicus* or *A. terreus* (Table 2). The most striking effect was observed using the secretome of *A. terreus*, which significantly decreased the amount of fucose, arabinose, xylose, galactose, glucose, and uronic acids compared to the Rovabio^®^ alone.

Based on the analysis of the sugar composition, we believe the observed boosting effect to be due to the solubilization of pectins. Soybean pectic polysaccharides are mainly composed by RGI [44,45,46,47], a complex polysaccharide composed of a backbone of alternating rhamnose and galacturonic acid residues with side chains containing galactose and/or arabinose residues. The structure of these side chains and the degree of substitution of rhamnose residues are extremely variable. Its complete hydrolysis requires the complementary action of more than 30 CAZymes [48]. Previous studies have shown that the Rovabio^®^ enzymatic cocktail displays a limited number of CAZymes acting on pectin, with only GH28 polygalacturonases, GH78 rhamnosidases, and GH53 endo-galactanases [24,26,27]. This suggests that the *Aspergilli* secretomes may contain specific CAZymes targetting pectin that are absent in the Rovabio^®^ cocktail. *Aspergilli* are well known for their ability to secrete pectinolytic enzymes [49,50,51,52]. For instance, *Aspergillus niger* has been studied under 16 different growth conditions to determine the role of the 26 genes encoding secreted pectinolytic enzymes [53]. It is therefore not surprising to find some *Aspergilli* strains with high potential for the degradation of SBM when supplemented with the Rovabio^®^ enzyme cocktail. However, this ability to degrade SBM does not apply to all *Aspergilli* strains tested in the frame of this study; as we demonstrated, *A. niger* (BRFM 280) and *A. brasiliensis* (BFRM 103) were not efficient to supplement the Rovabio^®^ (Figure S3).

### 3.7. Upscaling the Production of A. terreus Secretome in Bioreactor

**Figure 6:**
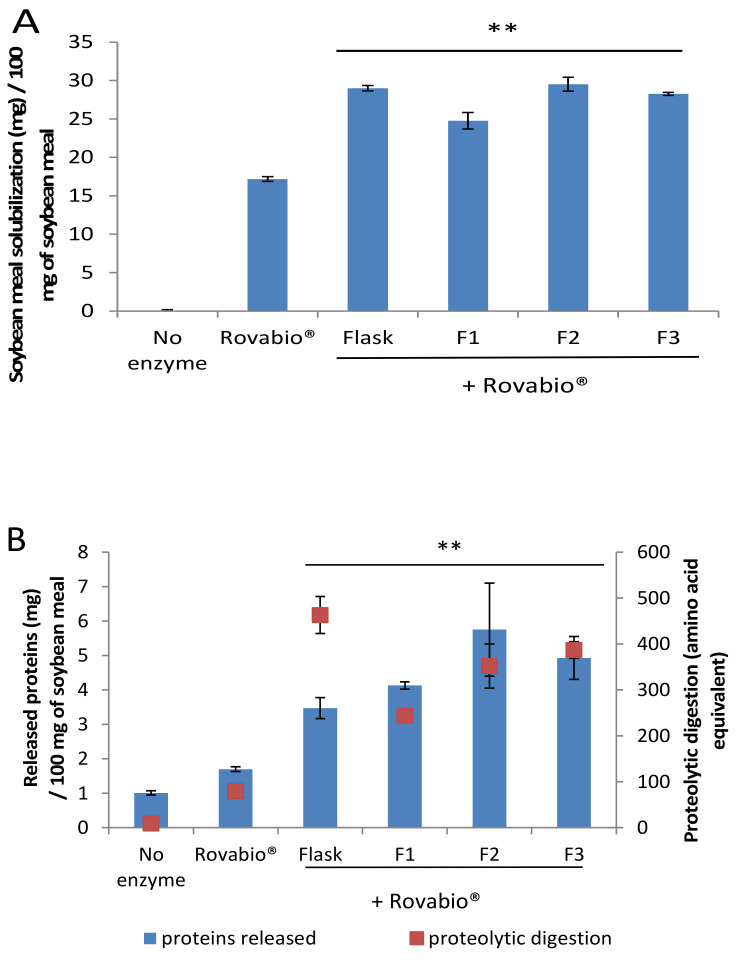

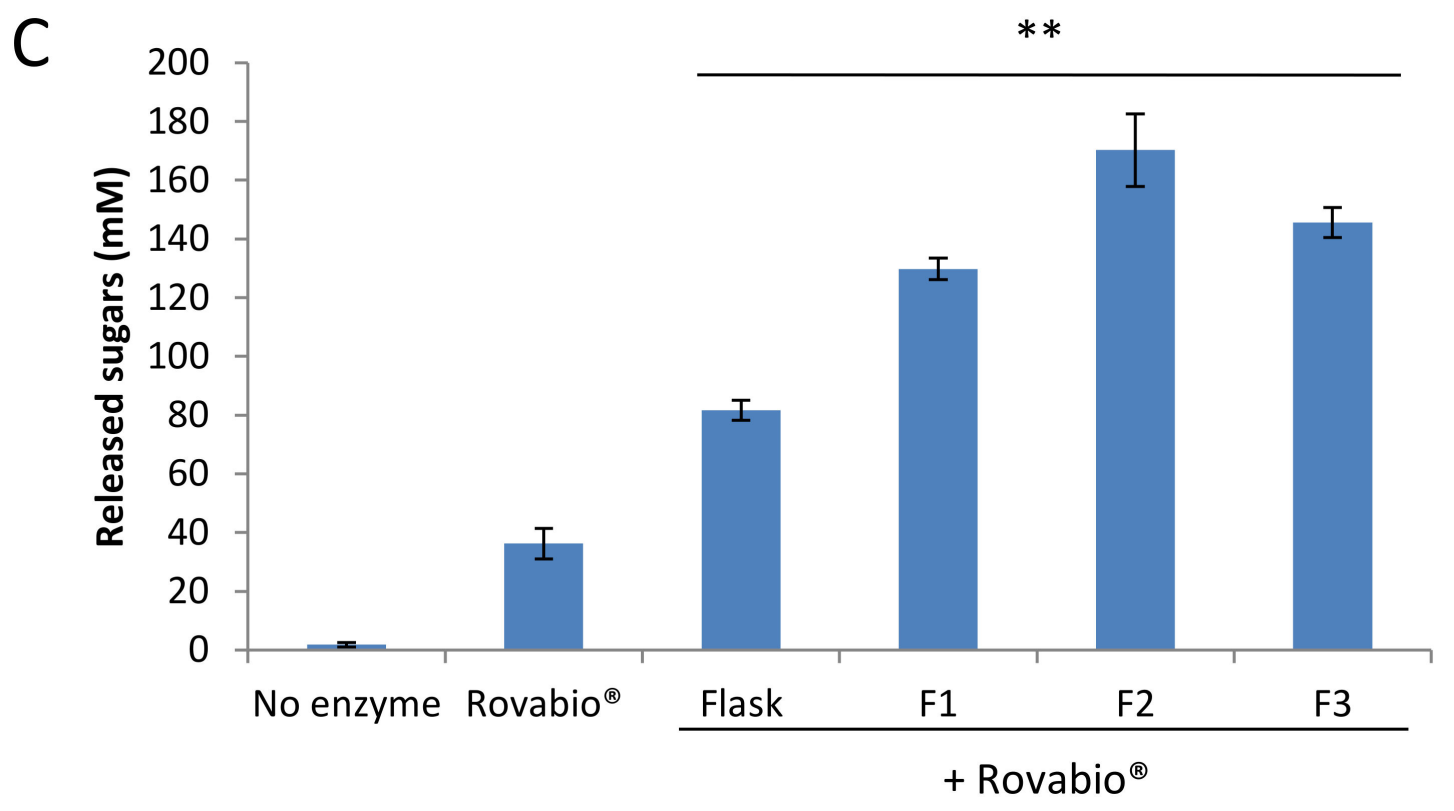
Effect of the *Aspergillus terreus* secretomes produced in bioreactor on the protein and sugars released from soybean meal. Panel (**A**): soybean meal solubilization after hydrolysis. F1, F2, and F3 are secretomes from *Aspergillus terreus* produced in bioreactors (experimental conditions in Table S2). Panel (**B**): total amount of released proteins (blue) and proteolytic digestion (red) after supplementation of Rovabio® with *A. terreus* secretomes produced in flask. Panel (**C**): soluble sugars released after SBM hydrolysis by Rovabio® supplemented with the *A. terreus* secretomes produced in bioreactor. Error bars indicate standard deviations of triplicate independent experiments. The significance of the results between Rovabio® condition and Rovabio® supplementation with secretomes (Flask and F1, F2, F3 bioreactors) was assessed using *t*-test (*n* = 3). The *p*-value obtained was <0.01 (**) for all the results.

To upscale and validate the supplementation effect of the *A. terreus* secretome, a bioreactor production was carried out. Three different 2-L bioreactors were prepared with variations in the number of spores inoculated and the size of the SBP particles (Table S2). The secretomes produced were collected and tested for their ability to supplement the Rovabio^®^ cocktail as previously described. All secretomes of *A. terreus* produced in bioreactor were able to improve SBM solubilization to the same extend as Rovabio^®^ supplemented by *A. terreus* produced in flask (Figure 6A). Of note, the supplementation using *A. terreus* secretomes produced in bioreactor F3 released more proteins (Figure 6B) than the secretomes produced in flask. Supplementation of Rovabio^®^ with all the different *A. terreus* secretomes produced in bioreactors released more sugars than the secretomes produced in flasks (Figure 6C). The fact that we managed to keep the boosting effect on SBM using *Aspergilli* secretomes produced in bioreactor is promising for further investigations at higher scale to attempt in vivo assays which require higher amount of enzymes [13,54].

## 4. Conclusions

In this study, we demonstrated the high potential of two *Aspergilli* strains, *A. japonicus* (BRFM 405) and *A. terreus* (BRFM 111), to upgrade the enzymatic cocktail Rovabio^®^ for SBM degradation. These fungal secretomes and their enzymes offer a promising solution to process industrial food coproducts into suitable animal feed solutions in the current context of circular economy. This study paves the way for future work aimed at identifying and characterizing the enzymes responsible for this improvement.

## Figures and Tables

**Table 1:** Fungal strains selected for the functional screening.

Species Name	BRFM Number	Phylum	Family
*Absidia glauca*	2463	Mucoromycota	*Cunninghamellaceae*
*Amauroderma calcigenum*	1190	Basidiomycota	*Ganodermataceae*
*Artolenzites elegans*	1280	Basidiomycota	*Polyporaceae*
*Aspergillus japonicus*	405	Ascomycota	*Trichocomaceae*
*Aspergillus terreus*	111	Ascomycota	*Trichocomaceae*
*Atheloderma mirabile*	2371	Basidiomycota	*Hymenochaetales*
*Bjerkandera adusta*	274	Basidiomycota	*Meruliaceae*
*Ceriporiopsis rivulosa*	663	Basidiomycota	*Phanerochaetaceae*
*Ceriporiopsis subvermispora*	394	Basidiomycota	*Phanerochaetaceae*
*Colletotrichum theobromicola*	1632	Ascomycota	*Glomerellaceae*
*Coniochaeta rubrosetosa*	1331	Ascomycota	*Coniochaetaceae*
*Coniophora arida*	494	Basidiomycota	*Coniophoraceae*
*Cookeina sulcipes*	2338	Ascomycota	*Sarcoscyphaceae*
*Crustomyces subabruptus*	793	Basidiomycota	*Cystostereaceae*
*Cyclocybe aegerita*	493	Basidiomycota	*Bolbitiaceae*
*Dichostereum effuscatum*	91	Basidiomycota	*Lachnocladiaceae*
*Eutypella scoparia*	1012	Ascomycota	*Diatrypaceae*
*Fomitiporia mediterranea*	2470	Basidiomycota	*Hymenochaetaceae*
*Fomitopsis pinicola*	886	Basidiomycota	*Formitopsidaceae*
*Gloeophyllum odoratum*	1454	Basidiomycota	*Gloephyllaceae*
*Gymnopilus junonius*	969	Basidiomycota	*Cortinariaceae*
*Hericium coralloides*	807	Basidiomycota	*Hericiaceae*
*Heterobasidion annosum*	238	Basidiomycota	*Bondarzewiaceae*
*Hypomyces luteovirens*	1580	Ascomycota	*Hypocreaceae*
*Laetisaria arvalis*	512	Basidiomycota	*Corticiaceae*
*Lentinellus castoreus*	668	Basidiomycota	*Auriscalpiaceae*
*Lentinula edodes*	353	Basidiomycota	*Marasmiaceae*
*Lepista nuda*	845	Basidiomycota	*Tricholomataceae*
*Lophiostoma arundinis*	1636	Ascomycota	*Lophiostomataceae*
*Macrolepiota fuliginosa*	851	Basidiomycota	*Agaricaceae*
*Mortierella alpina*	2447	Mucoromycota	*Morcierellaceae*
*Mycosphaerella lateris*	1628	Ascomycota	*Mycosphaerella*
*Nectria pseudotrichia*	1017	Ascomycota	*Nectriaceae*
*Neurospora crassa*	1092	Ascomycota	*Sordariaceae*
*Oxyporus latemarginatus*	678	Basidiomycota	*Meruliaceae*
*Peniophora albobadia*	788	Basidiomycota	*Peniophoraceae*
*Pestalotiopsis sp*	1648	Ascomycota	*Amphisphaeriaceae*
*Phaeosphaeria spartinicola*	1633	Ascomycota	*Phaeosphariaceae*
*Phanerochaete chrysosporium*	276	Basidiomycota	*Phanerochaetaceae*
*Phycomyces blakesleeanus*	1098	Mucoromycota	*Phycomycetaceae*
*Pleospora leptosphaerulinoides*	2474	Ascomycota	*Pleosporaceae*
*Pleurotus ostreatus*	853	Basidiomycota	*Pleurotaceae*
*Podospora anserina*	977	Ascomycota	*Lasiosphaeriaceae*
*Pycnoporus sanguineus*	902	Basidiomycota	*Polyporaceae*
*Rhizoctonia solani*	2454	Basidiomycota	*Ceratobasidiaceae*
*Rhizopus arrhizus var. arrhizus*	1095	Mucoromycota	*Mucoraceae*
*Sinosphaeria bambusicola*	1245	Ascomycota	*Thyrdiaceae*
*Sistotrema coroniferum*	803	Basidiomycota	*Hydnaceae*
*Xylobolus frustulatus*	768	Basidiomycota	*Stereaceae*

**Table 2:** Compositional analysis of the residual dry matter sugar content after hydrolysis. SBM was hydrolyzed with Rovabio^®^, supplemented or not with *Aspergillus terreus* or *Aspergillus japonicus* secretome grown on SBP (0.45 mg of enzyme). The residual dry matter sugar composition was analyzed. Rha: rhamnose, Fuc: fucose, Ara: arabinose, Xyl: Xylose, Man: mannose, Gal: galactose, Glc: glucose, and UA: uronic acids. Standard deviations of triplicate independent experiments are indicated. Significance of the results between Rovabio^®^ condition and Rovabio^®^ supplementation with secretome was assessed using *t*-test (*n* = 3) with *p*-value: * *p*-value < 0.05; ** *p*-value < 0.01, both indicated by an arrow.

Massic%			
	Rovabio^®^	Rovabio^®^ + *Aj*	Rovabio^®^ + *At*
Rha	0.315 ± 0.007	0.375 ± 0.026	0.315 ± 0.071
Fuc	0.450 ± 0.028	0.414 ± 0.360	0.298 ± 0.061 ↘ *
Ara	1.644 ± 0.199	1.030 ± 0.065	0.882 ± 0.106 ↘ **
Xyl	1.909 ± 0.070	2.220 ± 0.218	1.158 ± 0.269 ↘ *
Man	1.136 ± 0.102	0.915 ± 0.122	0.853 ± 0.165
Gal	1.742 ± 0.082	1.231 ± 0.104 ↘ **	0.794 ± 0.078 ↘ **
Glc	6.531 ± 0.841	5.288 ± 0.249	3.468 ± 0.544 ↘ **
UA	5.687 ± 0.206	7.154 ± 0.097	4.163 ± 0.334 ↘ **

## Data Availability

The data presented in this study are available in article and Supplementary Materials.

## Supplementary Materials

The following are available online at https://www.mdpi.com/article/10.3390/jof7040278/s1, Figure S1: Electrophoresis profiles of each secretomes used for Rovabio^®^ enzymatic cocktail supplementation. Figure S2: Solubilization of soybean meal using different amounts of Rovabio^®^. Figure S3: Enzymatic degradation of soybean meal by different strains of *Aspergilli*. Table S1: Protein content of each produced and concentrated secretomes for Rovabio^®^ enzymatic cocktail supplementation. Table S2: Presentation of different tested parameters in bioreactor experiments.
