# Supplementary material for: The Secretomes of Aspergillus japonicus and Aspergillus terreus Supplement the Rovabio® Enzyme Cocktail for the Degradation of Soybean Meal for Animal Feed
Source: J Fungi (Basel). 2021 Apr 7;7(4):278. doi: 10.3390/jof7040278 (PMC8067802; doi:10.3390/jof7040278)

Supplementary Figures and Tables

The secretomes of *Aspergillus japonicus* and *Aspergillus terreus* supplement the Rovabio® enzyme cocktail for the degradation of soybean meal for animal feed

Delphine Grandmontagne[1,2], David Navarro[1,3], Virginie Neugnot-Roux[2], Simon Ladevèze[1,4], Jean-Guy Berrin[1,*]

[1]INRAE, Aix Marseille Univ., BBF, UMR1163, 13009 Marseille, France

[2]ADISSEO, 135 avenue de Rangueil, INSA Toulouse, Hall Gilbert Durand, 31400 Toulouse, France

[3]CIRM-CF, INRAE, Aix Marseille Univ., 13009 Marseille, France

[4]Present address: University of Cambridge, Sanger Building, 80 Tennis Ct Rd, Cambridge CB2 1GA, UK

*Correspondence to: Jean-Guy Berrin (jean-guy.berrin@inrae.fr)

Supplementary Table 1: Protein content of each produced and concentrated secretomes for Rovabio® enzymatic cocktail supplementation. *Gymnopilus junonius* SBP data are missing as the secretome was too viscous and could not be collected and processed.

Fungal strain	Substrate	Protein concentration (mg/ml)
Absidia glauca	SBM	0.6
	SBP	0.4
Gymnopilus junonius	SBM	1.5
	SBP	-
Lentinula edodes	SBM	0.4
	SBP	0.5
Aspergillus terreus	SBM	3.4
	SBP	3.8
Aspergillus japonicus	SBM	4.3
	SBP	2.1

Supplementary Table 2: Presentation of different tested parameters in bioreactor experiments.

Bioreactor	Inoculum	Sugar Beet Particles Size
F1	2.10^8 spores/L	3mm
F2	2.10^8 spores/L	Micronized (<100 µm)
F3	2.10^9 spores/L	Micronized (<100 µm)

Supplementary Figure 1 : Electrophoresis profiles of each secretomes used for Rovabio® enzymatic cocktail supplementation. 10 µg of protein from each secretome were loaded onto a 15% Tris Glycine SDS-PAGE, and proteins were stained with Coomassie Blue. The molecular marker (Page Ruler) (M) are indicated on the left.
Secretomes of *Aspergillus terreus* SBM (lane 1), *Aspergillus terreus* SBP (lane 2), *Aspergillus japonicas* SBM (lane 3), *Aspergillus japonicus* SBP (lane 4), *Absidia glauca* SBM (lane 5), *Absidia glauca* SBP (lane 6), *Gymnopilus junonius* SBM (lane 7), *Lentinula edodes* SBM (lane 8) and *Lentinula edodes* SBP (lane 9) are shown.

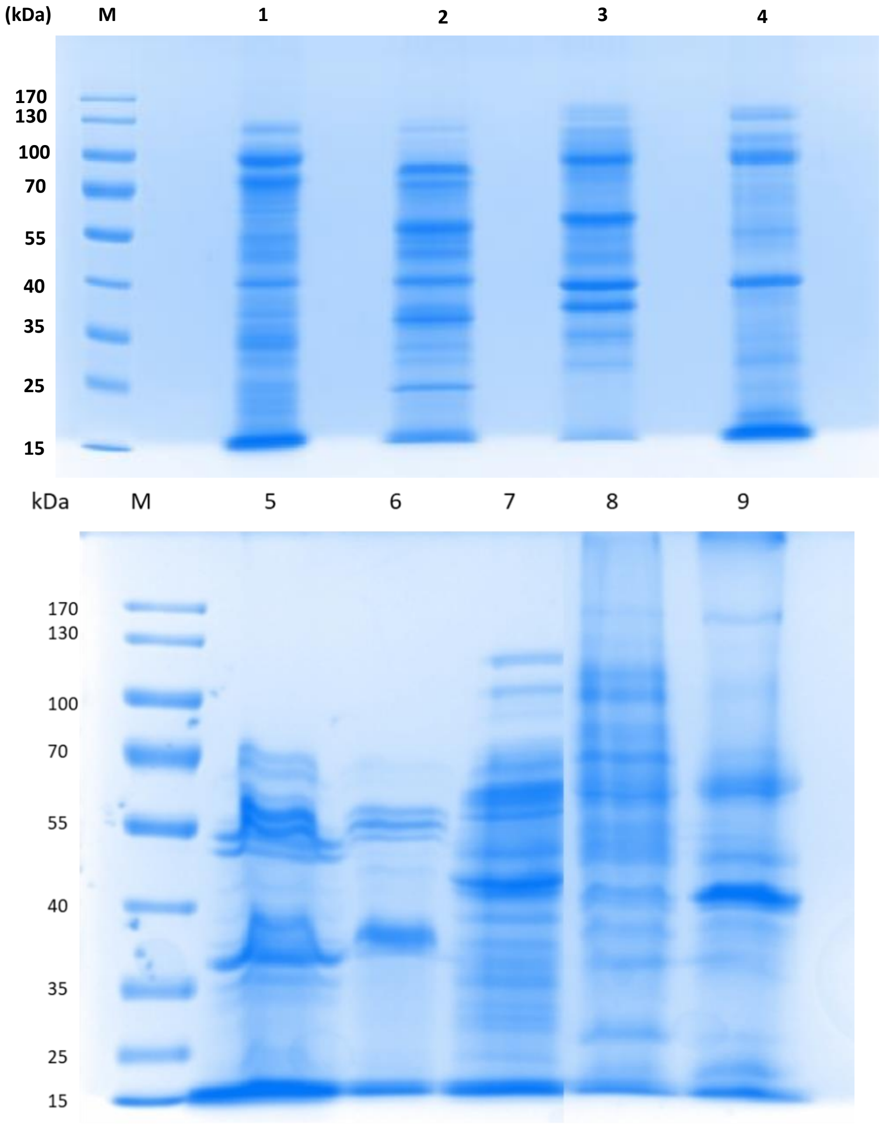

Supplementary Figure 2: Solubilization of soybean meal using different amounts of Rovabio®. The amount of protein was quantified using the Bradford assay.

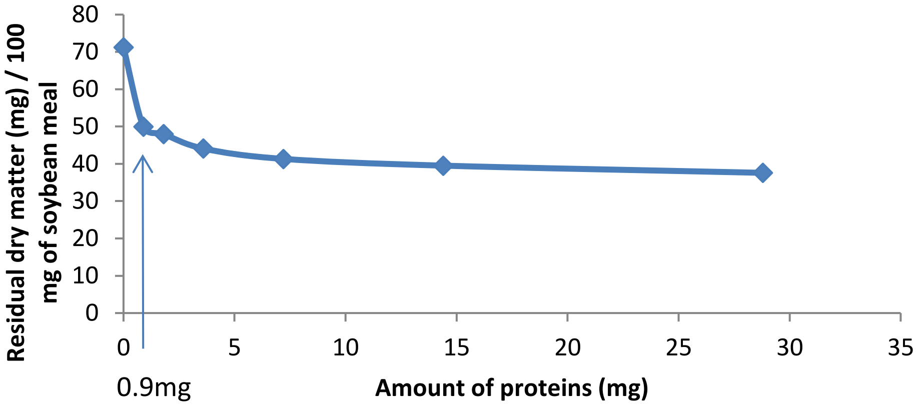

Supplementary Figure 3: Enzymatic degradation of soybean meal by different strains of *Aspergilli*. Rovabio® (0.52 mg) is supplemented with 0.55 mg of secreted proteins from different strains of *Aspergilli*, cultured on SBM (Soybean Meal) or SBP (Sugar Beet Pulp). An : *Aspergillus niger* (BRFM 280); Aj: *Aspergillus japonicus* (BRFM 405) ; At: *Aspergillus terreus* (BRFM 111) ; Ab: *Aspergillus brasiliensis* (BRFM 103); Atu: *Aspergillus tubengensis* (BRFM 1521). Significance between the results was assessed using t-test (n=3) with *p* value: *, *p* value < 0.05; ** *p* value < 0.01.

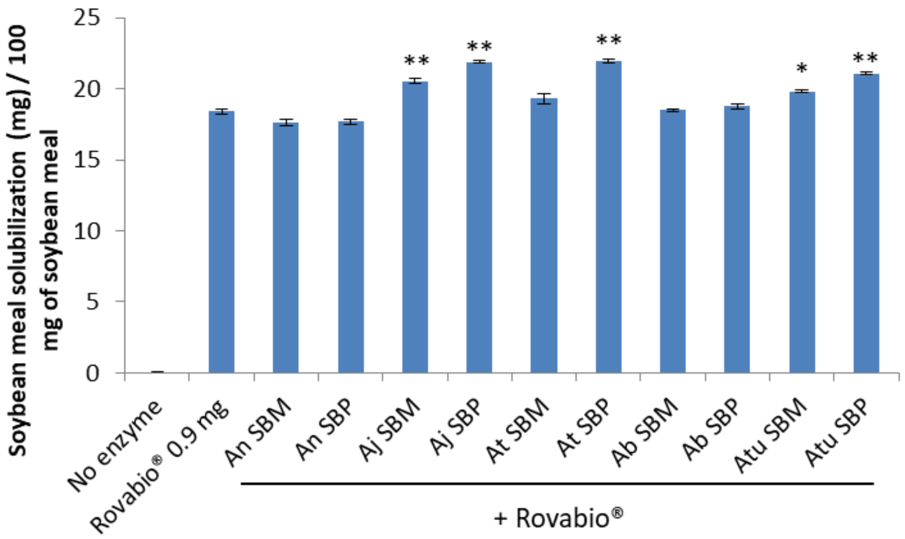

Supplement: Supplementary file 1 [file jof-07-00278-s001.pdf]
